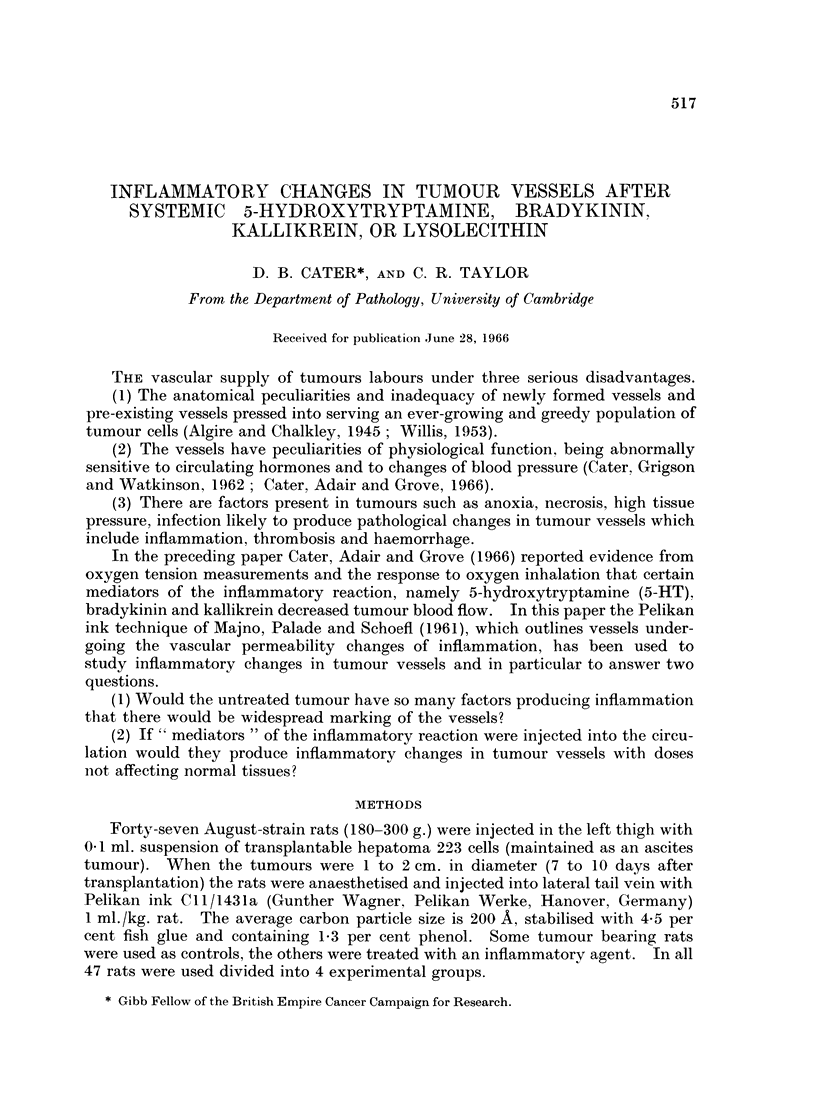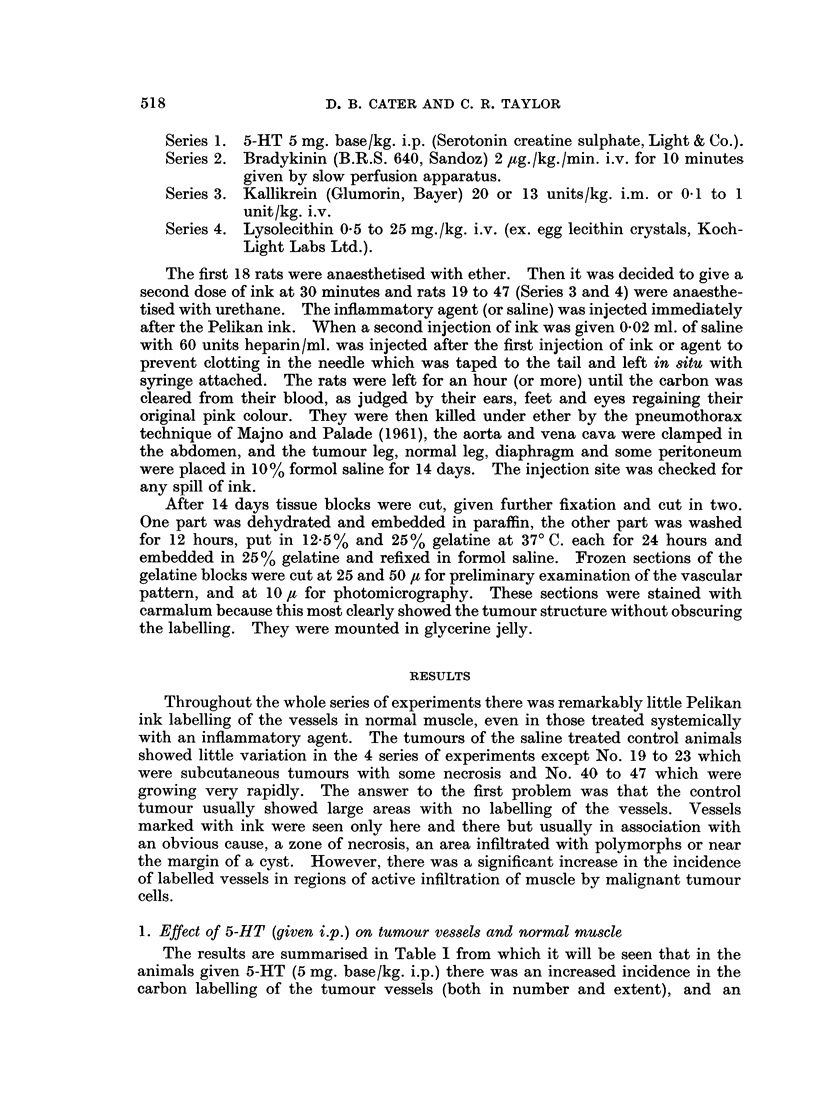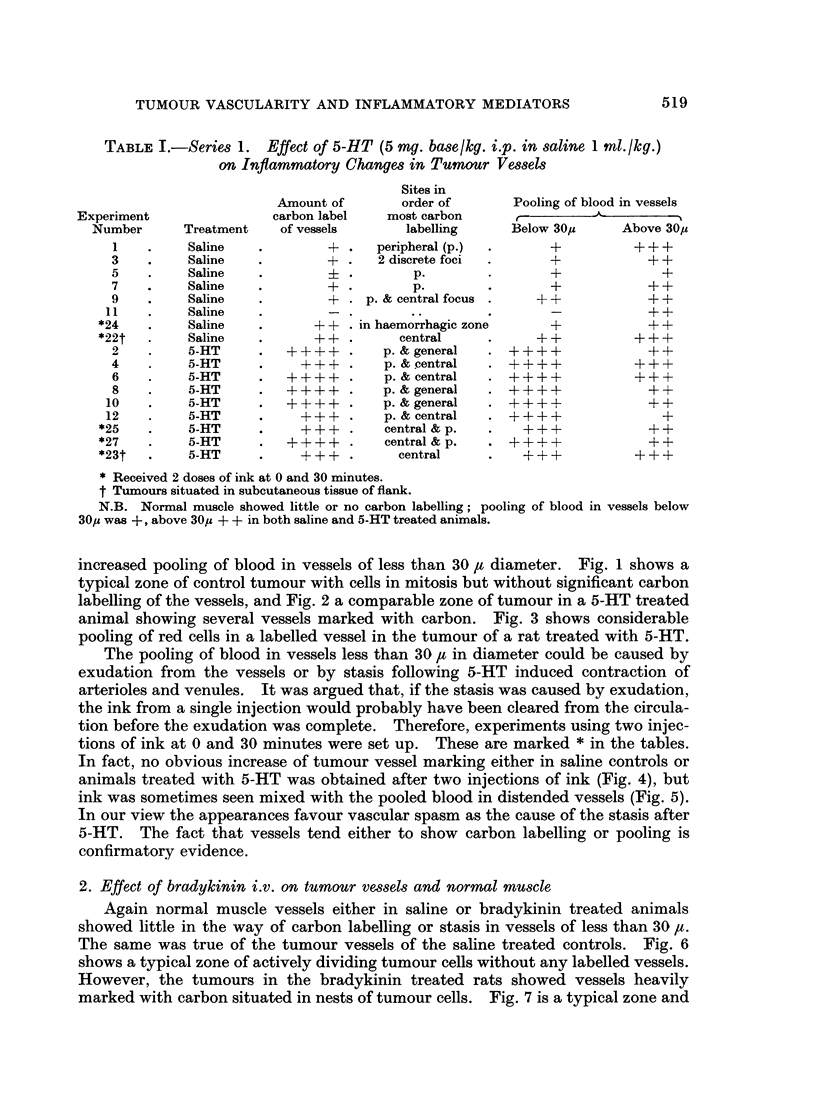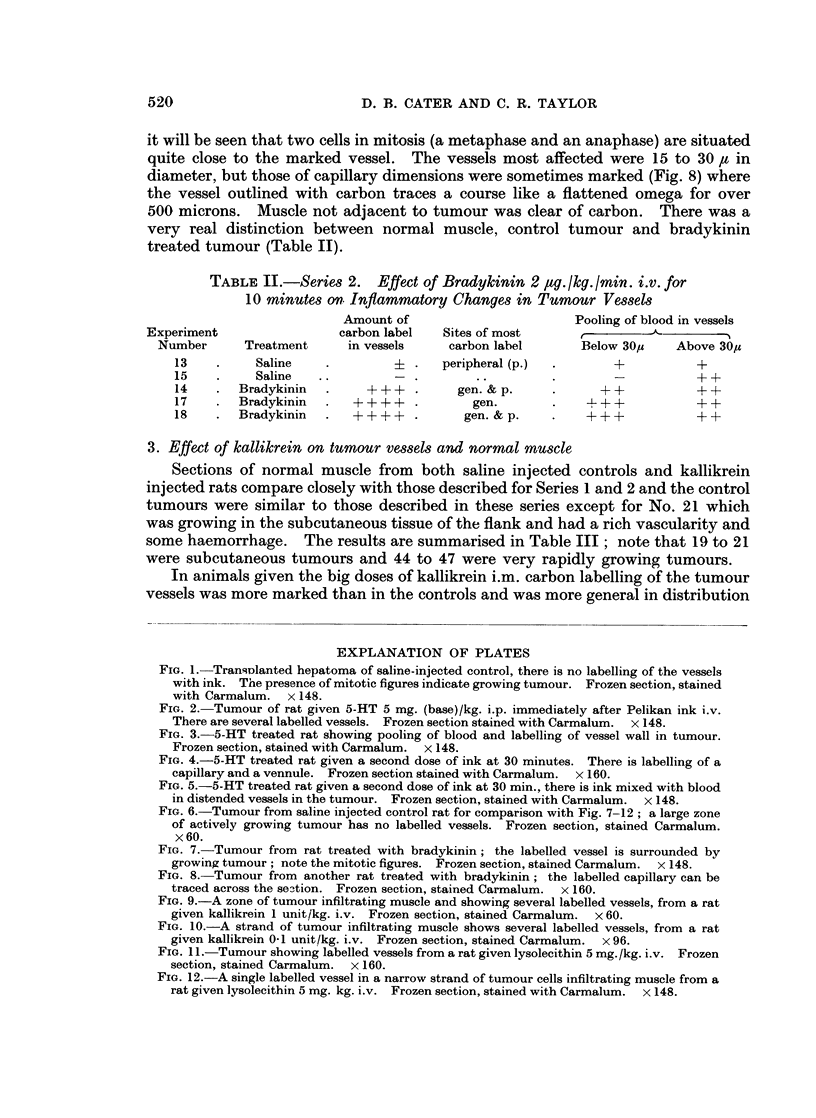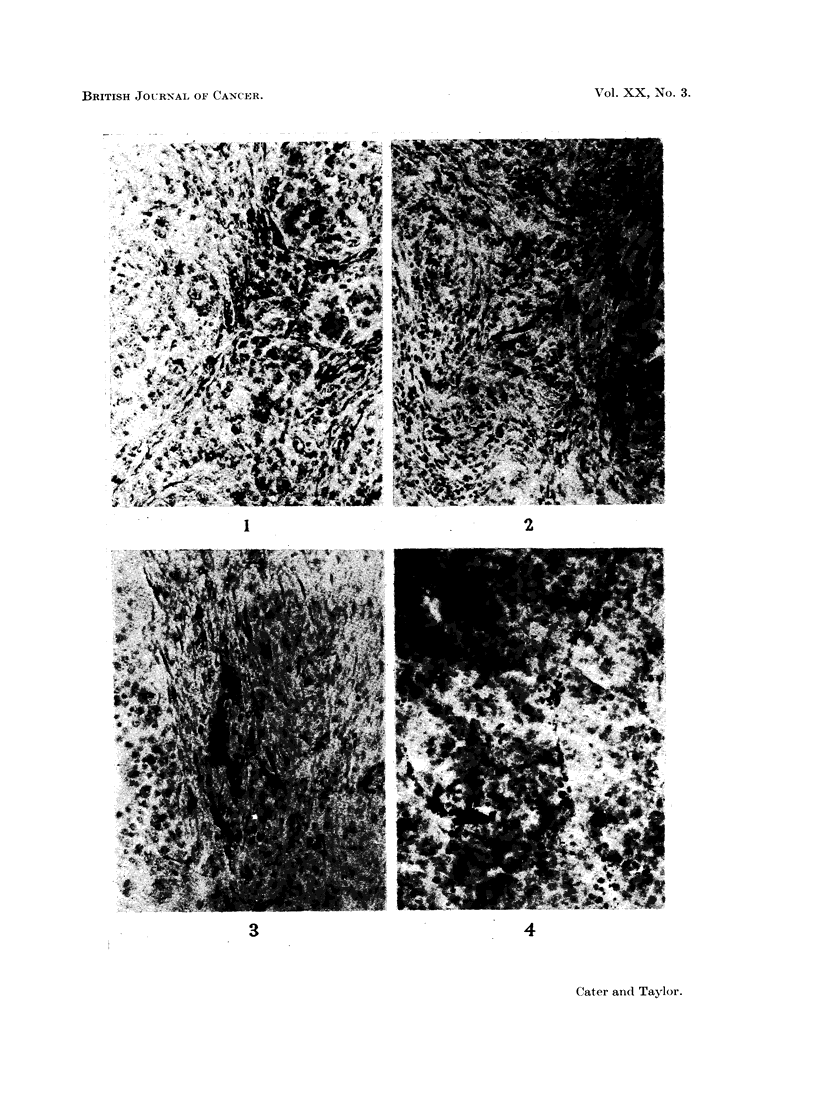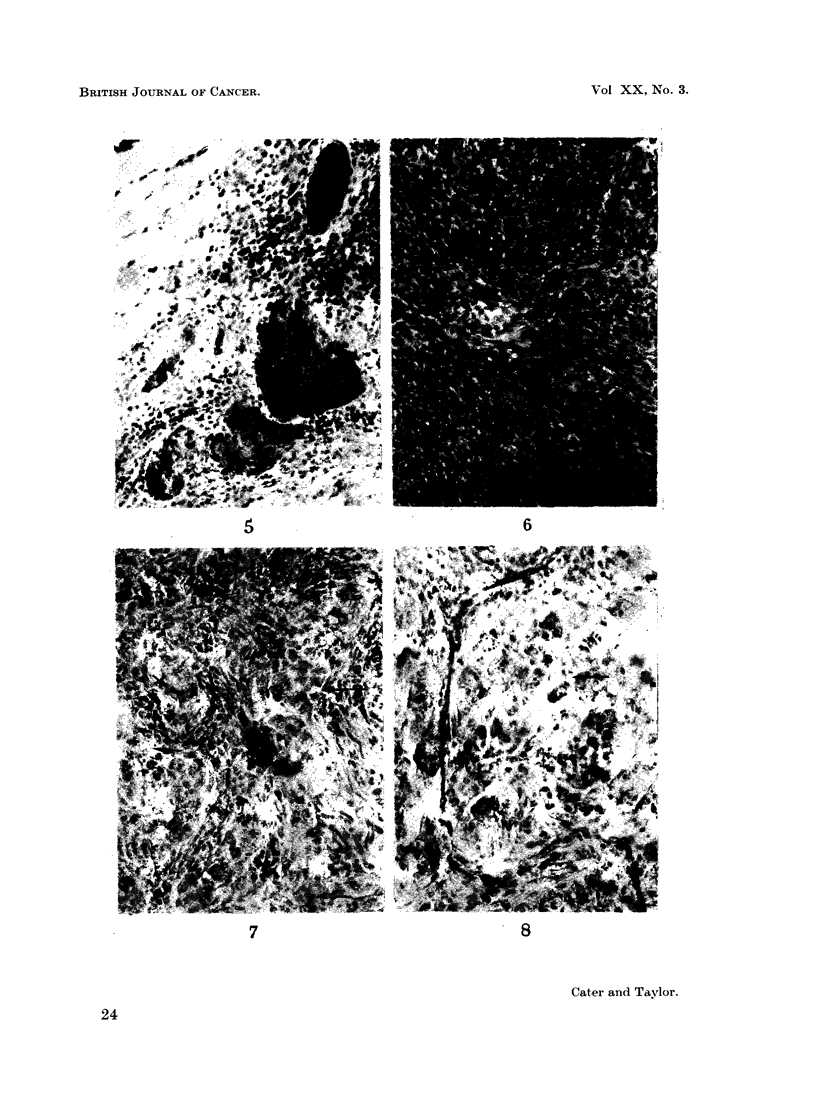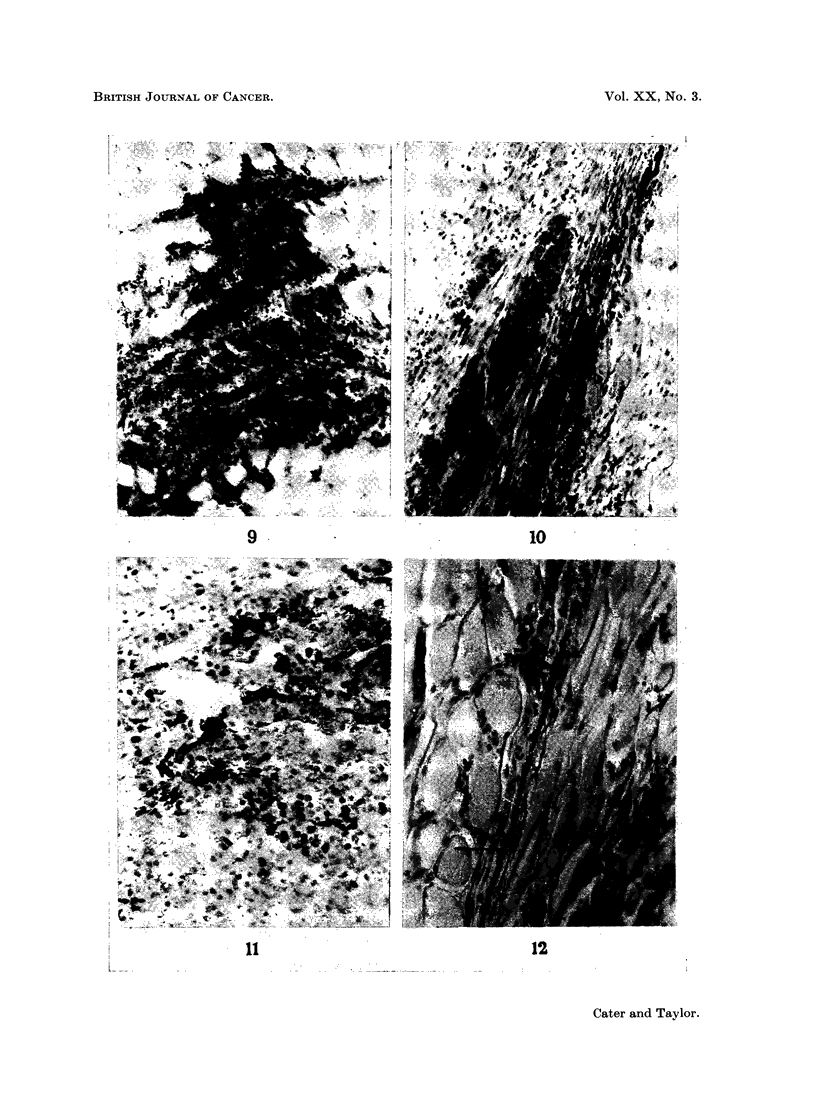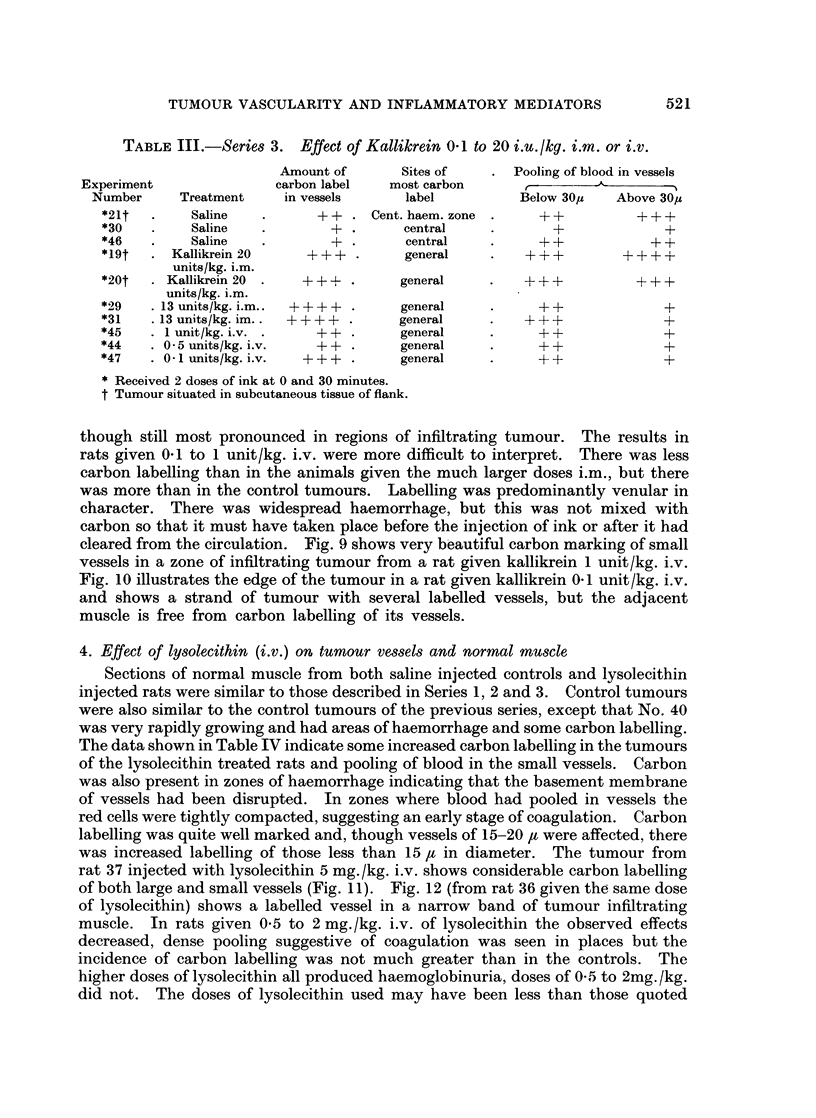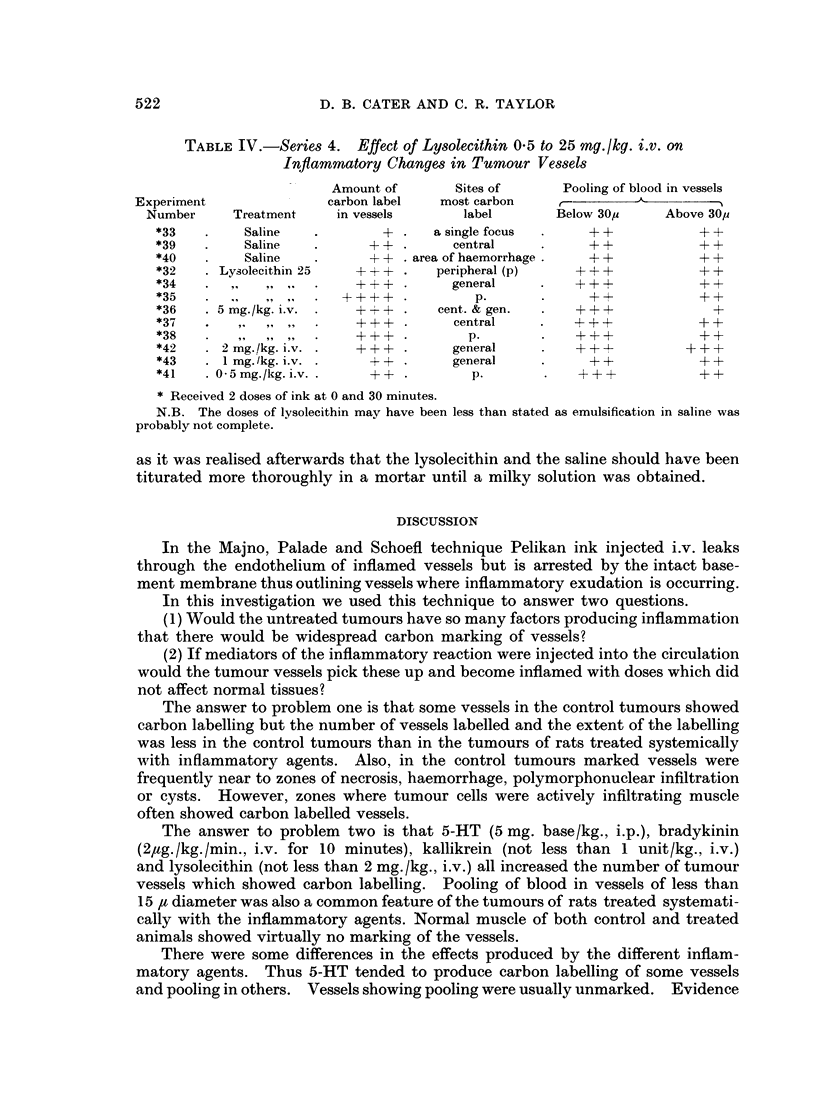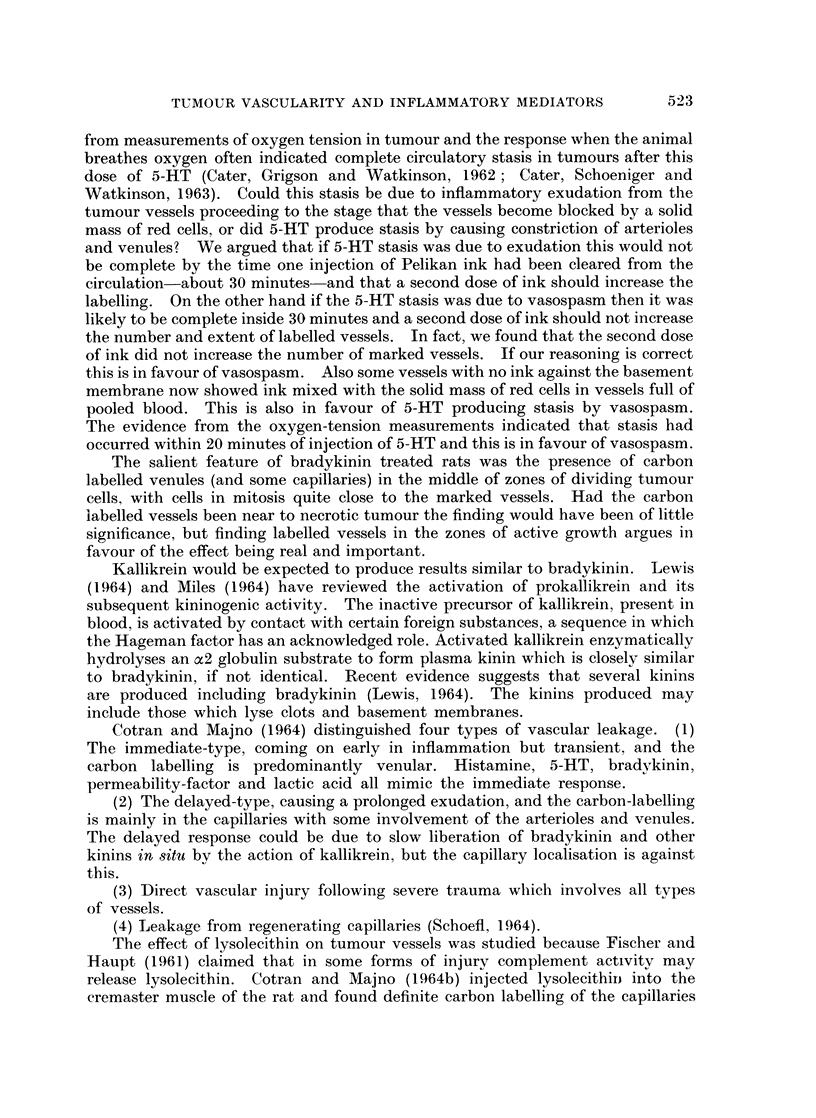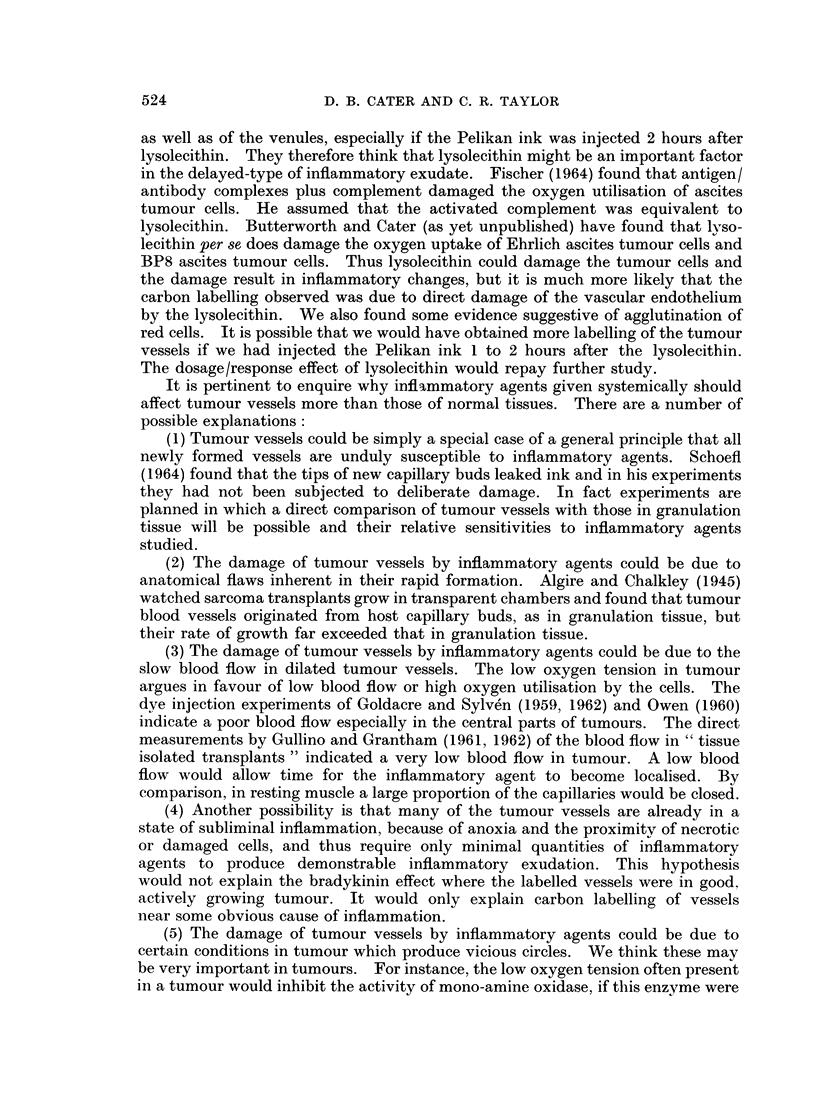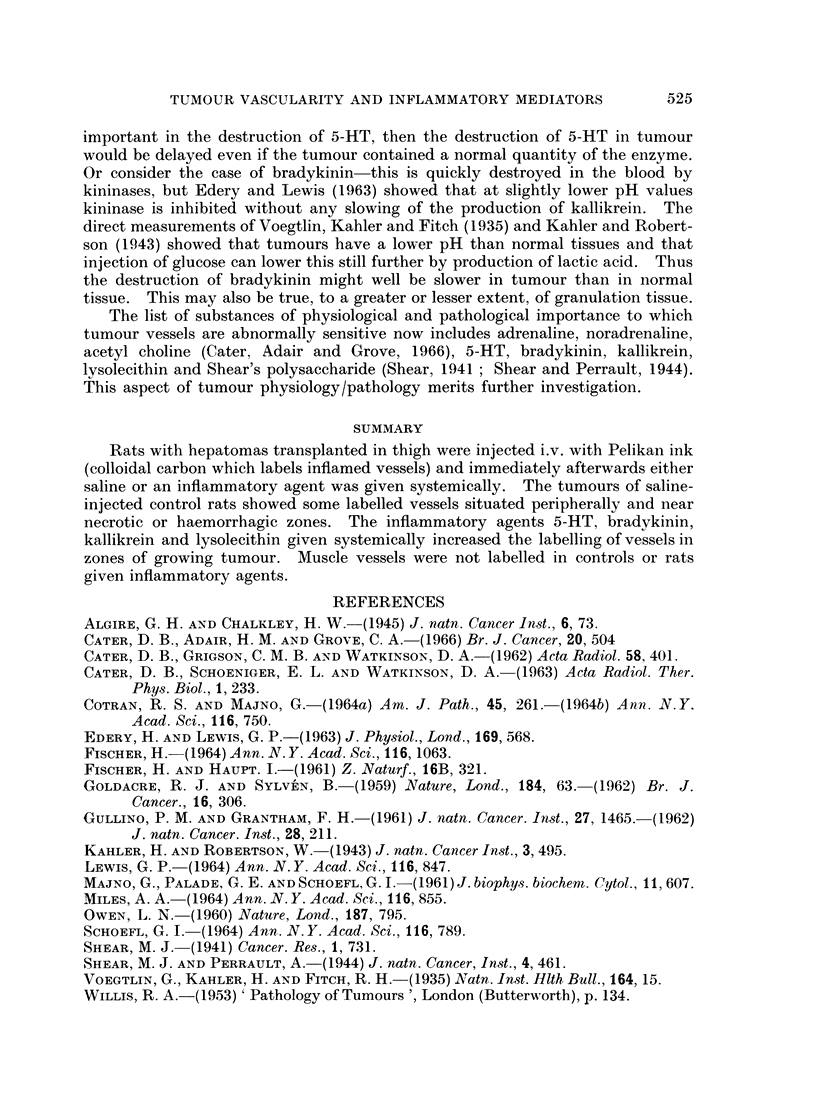# Inflammatory changes in tumour vessels after systemic 5-hydroxytryptamine, bradykinin, kallikrein, or lysolecithin.

**DOI:** 10.1038/bjc.1966.63

**Published:** 1966-09

**Authors:** D. B. Cater, C. R. Taylor

## Abstract

**Images:**


### INFLAMMATORY CHANGES IN TUMOUR VESSELS AFTER SYSTEMIC 5-HYDROXYTRYPTAMINE, BRADYKININ, KALLIKREIN, OR LYSOLECITHIN

D. B. CATER*, AND C. R. TAYLOR

*From the Department of Pathology, University of Cambridge*

Received for publication June 28, 1966

THE vascular supply of tumours labours under three serious disadvantages.

(1) The anatomical peculiarities and inadequacy of newly formed vessels and pre-existing vessels pressed into serving an ever-growing and greedy population of tumour cells (Algire and Chalkley, 1945 ; Willis, 1953).

(2) The vessels have peculiarities of physiological function, being abnormally sensitive to circulating hormones and to changes of blood pressure (Cater, Grigson and Watkinson, 1962 ; Cater, Adair and Grove, 1966).

(3) There are factors present in tumours such as anoxia, necrosis, high tissue pressure, infection likely to produce pathological changes in tumour vessels which include inflammation, thrombosis and haemorrhage.

In the preceding paper Cater, Adair and Grove (1966) reported evidence from oxygen tension measurements and the response to oxygen inhalation that certain mediators of the inflammatory reaction, namely 5-hydroxytryptamine (5-HT), bradykinin and kallikrein decreased tumour blood flow. In this paper the Pelikan ink technique of Majno, Palade and Schoefl (1961), which outlines vessels undergoing the vascular permeability changes of inflammation, has been used to study inflammatory changes in tumour vessels and in particular to answer two questions.

(1) Would the untreated tumour have so many factors producing inflammation that there would be widespread marking of the vessels?

(2) If " mediators " of the inflammatory reaction were injected into the circulation would they produce inflammatory changes in tumour vessels with doses not affecting normal tissues?

### METHODS

Forty-seven August-strain rats (180–300 g.) were injected in the left thigh with 0·1 ml. suspension of transplantable hepatoma 223 cells (maintained as an ascites tumour). When the tumours were 1 to 2 cm. in diameter (7 to 10 days after transplantation) the rats were anaesthetised and injected into lateral tail vein with Pelikan ink C11/1431a (Gunther Wagner, Pelikan Werke, Hanover, Germany) 1 ml./kg. rat. The average carbon particle size is 200 Å, stabilised with 4·5 per cent fish glue and containing 1·3 per cent phenol. Some tumour bearing rats were used as controls, the others were treated with an inflammatory agent. In all 47 rats were used divided into 4 experimental groups.

* Gibb Fellow of the British Empire Cancer Campaign for Research.

Series 1. 5-HT 5 mg. base/kg. i.p. (Serotonin creatine sulphate, Light & Co.).
Series 2. Bradykinin (B.R.S. 640, Sandoz) 2 $\mu$g./kg./min. i.v. for 10 minutes given by slow perfusion apparatus.
Series 3. Kallikrein (Glumorin, Bayer) 20 or 13 units/kg. i.m. or 0·1 to 1 unit/kg. i.v.
Series 4. Lysolecithin 0·5 to 25 mg./kg. i.v. (ex. egg lecithin crystals, Koch-Light Labs Ltd.).

The first 18 rats were anaesthetised with ether. Then it was decided to give a second dose of ink at 30 minutes and rats 19 to 47 (Series 3 and 4) were anaesthetised with urethane. The inflammatory agent (or saline) was injected immediately after the Pelikan ink. When a second injection of ink was given 0·02 ml. of saline with 60 units heparin/ml. was injected after the first injection of ink or agent to prevent clotting in the needle which was taped to the tail and left *in situ* with syringe attached. The rats were left for an hour (or more) until the carbon was cleared from their blood, as judged by their ears, feet and eyes regaining their original pink colour. They were then killed under ether by the pneumothorax technique of Majno and Palade (1961), the aorta and vena cava were clamped in the abdomen, and the tumour leg, normal leg, diaphragm and some peritoneum were placed in 10% formol saline for 14 days. The injection site was checked for any spill of ink.

After 14 days tissue blocks were cut, given further fixation and cut in two. One part was dehydrated and embedded in paraffin, the other part was washed for 12 hours, put in 12·5% and 25% gelatine at 37° C. each for 24 hours and embedded in 25% gelatine and refixed in formol saline. Frozen sections of the gelatine blocks were cut at 25 and 50 $\mu$ for preliminary examination of the vascular pattern, and at 10 $\mu$ for photomicrography. These sections were stained with carmalum because this most clearly showed the tumour structure without obscuring the labelling. They were mounted in glycerine jelly.

### RESULTS

Throughout the whole series of experiments there was remarkably little Pelikan ink labelling of the vessels in normal muscle, even in those treated systemically with an inflammatory agent. The tumours of the saline treated control animals showed little variation in the 4 series of experiments except No. 19 to 23 which were subcutaneous tumours with some necrosis and No. 40 to 47 which were growing very rapidly. The answer to the first problem was that the control tumour usually showed large areas with no labelling of the vessels. Vessels marked with ink were seen only here and there but usually in association with an obvious cause, a zone of necrosis, an area infiltrated with polymorphs or near the margin of a cyst. However, there was a significant increase in the incidence of labelled vessels in regions of active infiltration of muscle by malignant tumour cells.

### 1. *Effect of* 5-*HT* (*given i.p.*) *on tumour vessels and normal muscle*

The results are summarised in Table I from which it will be seen that in the animals given 5-HT (5 mg. base/kg. i.p.) there was an increased incidence in the carbon labelling of the tumour vessels (both in number and extent), and an

TABLE I.—*Series 1. Effect of 5-HT (5 mg. base/kg. i.p. in saline 1 ml./kg.) on Inflammatory Changes in Tumour Vessels*

| Experiment Number | Treatment | Amount of carbon label of vessels | Sites in order of most carbon labelling | Pooling of blood in vessels: Below 30μ | Above 30μ |
|---|---|---|---|---|---|
| 1 | Saline | + | peripheral (p.) | + | +++ |
| 3 | Saline | + | 2 discrete foci | + | ++ |
| 5 | Saline | ± | p. | + | + |
| 7 | Saline | + | p. | + | ++ |
| 9 | Saline | + | p. & central focus | ++ | ++ |
| 11 | Saline | – | .. | – | ++ |
| *24 | Saline | ++ | in haemorrhagic zone | + | ++ |
| *22† | Saline | ++ | central | ++ | +++ |
| 2 | 5-HT | ++++ | p. & general | ++++ | ++ |
| 4 | 5-HT | +++ | p. & central | ++++ | +++ |
| 6 | 5-HT | ++++ | p. & central | ++++ | +++ |
| 8 | 5-HT | ++++ | p. & general | ++++ | ++ |
| 10 | 5-HT | ++++ | p. & general | ++++ | ++ |
| 12 | 5-HT | +++ | p. & central | ++++ | + |
| *25 | 5-HT | +++ | central & p. | +++ | ++ |
| *27 | 5-HT | ++++ | central & p. | ++++ | ++ |
| *23† | 5-HT | +++ | central | +++ | +++ |

* Received 2 doses of ink at 0 and 30 minutes.
† Tumours situated in subcutaneous tissue of flank.
N.B. Normal muscle showed little or no carbon labelling; pooling of blood in vessels below 30μ was +, above 30μ ++ in both saline and 5-HT treated animals.

increased pooling of blood in vessels of less than 30 μ diameter. Fig. 1 shows a typical zone of control tumour with cells in mitosis but without significant carbon labelling of the vessels, and Fig. 2 a comparable zone of tumour in a 5-HT treated animal showing several vessels marked with carbon. Fig. 3 shows considerable pooling of red cells in a labelled vessel in the tumour of a rat treated with 5-HT.

The pooling of blood in vessels less than 30 μ in diameter could be caused by exudation from the vessels or by stasis following 5-HT induced contraction of arterioles and venules. It was argued that, if the stasis was caused by exudation, the ink from a single injection would probably have been cleared from the circulation before the exudation was complete. Therefore, experiments using two injections of ink at 0 and 30 minutes were set up. These are marked * in the tables. In fact, no obvious increase of tumour vessel marking either in saline controls or animals treated with 5-HT was obtained after two injections of ink (Fig. 4), but ink was sometimes seen mixed with the pooled blood in distended vessels (Fig. 5). In our view the appearances favour vascular spasm as the cause of the stasis after 5-HT. The fact that vessels tend either to show carbon labelling or pooling is confirmatory evidence.

2. *Effect of bradykinin i.v. on tumour vessels and normal muscle*

Again normal muscle vessels either in saline or bradykinin treated animals showed little in the way of carbon labelling or stasis in vessels of less than 30 μ. The same was true of the tumour vessels of the saline treated controls. Fig. 6 shows a typical zone of actively dividing tumour cells without any labelled vessels. However, the tumours in the bradykinin treated rats showed vessels heavily marked with carbon situated in nests of tumour cells. Fig. 7 is a typical zone and it will be seen that two cells in mitosis (a metaphase and an anaphase) are situated quite close to the marked vessel. The vessels most affected were 15 to 30 $\mu$ in diameter, but those of capillary dimensions were sometimes marked (Fig. 8) where the vessel outlined with carbon traces a course like a flattened omega for over 500 microns. Muscle not adjacent to tumour was clear of carbon. There was a very real distinction between normal muscle, control tumour and bradykinin treated tumour (Table II).

TABLE II.—*Series 2. Effect of Bradykinin 2 µg./kg./min. i.v. for 10 minutes on Inflammatory Changes in Tumour Vessels*

| Experiment Number | Treatment | Amount of carbon label in vessels | Sites of most carbon label | Pooling of blood in vessels | |
|---|---|---|---|---|---|
| | | | | Below 30$\mu$ | Above 30$\mu$ |
| 13 | Saline | ± | peripheral (p.) | + | + |
| 15 | Saline | – | .. | – | ++ |
| 14 | Bradykinin | +++ | gen. & p. | ++ | ++ |
| 17 | Bradykinin | ++++ | gen. | +++ | ++ |
| 18 | Bradykinin | ++++ | gen. & p. | +++ | ++ |

3. *Effect of kallikrein on tumour vessels and normal muscle*

Sections of normal muscle from both saline injected controls and kallikrein injected rats compare closely with those described for Series 1 and 2 and the control tumours were similar to those described in these series except for No. 21 which was growing in the subcutaneous tissue of the flank and had a rich vascularity and some haemorrhage. The results are summarised in Table III ; note that 19 to 21 were subcutaneous tumours and 44 to 47 were very rapidly growing tumours.

In animals given the big doses of kallikrein i.m. carbon labelling of the tumour vessels was more marked than in the controls and was more general in distribution

EXPLANATION OF PLATES

FIG. 1.—Transplanted hepatoma of saline-injected control, there is no labelling of the vessels with ink. The presence of mitotic figures indicate growing tumour. Frozen section, stained with Carmalum. ×148.

FIG. 2.—Tumour of rat given 5-HT 5 mg. (base)/kg. i.p. immediately after Pelikan ink i.v. There are several labelled vessels. Frozen section stained with Carmalum. ×148.

FIG. 3.—5-HT treated rat showing pooling of blood and labelling of vessel wall in tumour. Frozen section, stained with Carmalum. ×148.

FIG. 4.—5-HT treated rat given a second dose of ink at 30 minutes. There is labelling of a capillary and a vennule. Frozen section stained with Carmalum. ×160.

FIG. 5.—5-HT treated rat given a second dose of ink at 30 min., there is ink mixed with blood in distended vessels in the tumour. Frozen section, stained with Carmalum. ×148.

FIG. 6.—Tumour from saline injected control rat for comparison with Fig. 7–12 ; a large zone of actively growing tumour has no labelled vessels. Frozen section, stained Carmalum. ×60.

FIG. 7.—Tumour from rat treated with bradykinin ; the labelled vessel is surrounded by growing tumour ; note the mitotic figures. Frozen section, stained Carmalum. ×148.

FIG. 8.—Tumour from another rat treated with bradykinin ; the labelled capillary can be traced across the section. Frozen section, stained Carmalum. ×160.

FIG. 9.—A zone of tumour infiltrating muscle and showing several labelled vessels, from a rat given kallikrein 1 unit/kg. i.v. Frozen section, stained Carmalum. ×60.

FIG. 10.—A strand of tumour infiltrating muscle shows several labelled vessels, from a rat given kallikrein 0·1 unit/kg. i.v. Frozen section, stained Carmalum. ×96.

FIG. 11.—Tumour showing labelled vessels from a rat given lysolecithin 5 mg./kg. i.v. Frozen section, stained Carmalum. ×160.

FIG. 12.—A single labelled vessel in a narrow strand of tumour cells infiltrating muscle from a rat given lysolecithin 5 mg. kg. i.v. Frozen section, stained with Carmalum. ×148.

1

2

3

4

Cater and Taylor.

5

6

7

8

9

10

11

12

Cater and Taylor.

TABLE III.—*Series* 3. *Effect of Kallikrein* 0·1 *to* 20 *i.u./kg. i.m. or i.v.*

| Experiment Number | Treatment | Amount of carbon label in vessels | Sites of most carbon label | Pooling of blood in vessels: Below 30μ | Pooling of blood in vessels: Above 30μ |
|---|---|---|---|---|---|
| *21† | Saline | ++ | Cent. haem. zone | ++ | +++ |
| *30 | Saline | + | central | + | + |
| *46 | Saline | + | central | ++ | ++ |
| *19† | Kallikrein 20 units/kg. i.m. | +++ | general | +++ | ++++ |
| *20† | Kallikrein 20 units/kg. i.m. | +++ | general | +++ | +++ |
| *29 | 13 units/kg. i.m.. | ++++ | general | ++ | + |
| *31 | 13 units/kg. im.. | ++++ | general | +++ | + |
| *45 | 1 unit/kg. i.v. | ++ | general | ++ | + |
| *44 | 0·5 units/kg. i.v. | ++ | general | ++ | + |
| *47 | 0·1 units/kg. i.v. | +++ | general | ++ | + |

* Received 2 doses of ink at 0 and 30 minutes.
† Tumour situated in subcutaneous tissue of flank.

though still most pronounced in regions of infiltrating tumour. The results in rats given 0·1 to 1 unit/kg. i.v. were more difficult to interpret. There was less carbon labelling than in the animals given the much larger doses i.m., but there was more than in the control tumours. Labelling was predominantly venular in character. There was widespread haemorrhage, but this was not mixed with carbon so that it must have taken place before the injection of ink or after it had cleared from the circulation. Fig. 9 shows very beautiful carbon marking of small vessels in a zone of infiltrating tumour from a rat given kallikrein 1 unit/kg. i.v. Fig. 10 illustrates the edge of the tumour in a rat given kallikrein 0·1 unit/kg. i.v. and shows a strand of tumour with several labelled vessels, but the adjacent muscle is free from carbon labelling of its vessels.

4. *Effect of lysolecithin* (*i.v.*) *on tumour vessels and normal muscle*

Sections of normal muscle from both saline injected controls and lysolecithin injected rats were similar to those described in Series 1, 2 and 3. Control tumours were also similar to the control tumours of the previous series, except that No. 40 was very rapidly growing and had areas of haemorrhage and some carbon labelling. The data shown in Table IV indicate some increased carbon labelling in the tumours of the lysolecithin treated rats and pooling of blood in the small vessels. Carbon was also present in zones of haemorrhage indicating that the basement membrane of vessels had been disrupted. In zones where blood had pooled in vessels the red cells were tightly compacted, suggesting an early stage of coagulation. Carbon labelling was quite well marked and, though vessels of 15–20 μ were affected, there was increased labelling of those less than 15 μ in diameter. The tumour from rat 37 injected with lysolecithin 5 mg./kg. i.v. shows considerable carbon labelling of both large and small vessels (Fig. 11). Fig. 12 (from rat 36 given the same dose of lysolecithin) shows a labelled vessel in a narrow band of tumour infiltrating muscle. In rats given 0·5 to 2 mg./kg. i.v. of lysolecithin the observed effects decreased, dense pooling suggestive of coagulation was seen in places but the incidence of carbon labelling was not much greater than in the controls. The higher doses of lysolecithin all produced haemoglobinuria, doses of 0·5 to 2mg./kg. did not. The doses of lysolecithin used may have been less than those quoted

TABLE IV.—*Series 4. Effect of Lysolecithin 0·5 to 25 mg./kg. i.v. on Inflammatory Changes in Tumour Vessels*

| Experiment Number | Treatment | Amount of carbon label in vessels | Sites of most carbon label | Pooling of blood in vessels | |
|---|---|---|---|---|---|
| | | | | Below 30μ | Above 30μ |
| *33 | Saline | + | a single focus | ++ | ++ |
| *39 | Saline | ++ | central | ++ | ++ |
| *40 | Saline | ++ | area of haemorrhage | ++ | ++ |
| *32 | Lysolecithin 25 | +++ | peripheral (p) | +++ | ++ |
| *34 | ,, ,, ,, | +++ | general | +++ | ++ |
| *35 | ,, ,, ,, | ++++ | p. | ++ | ++ |
| *36 | 5 mg./kg. i.v. | +++ | cent. & gen. | +++ | + |
| *37 | ,, ,, ,, | +++ | central | +++ | ++ |
| *38 | ,, ,, ,, | +++ | p. | +++ | ++ |
| *42 | 2 mg./kg. i.v. | +++ | general | +++ | +++ |
| *43 | 1 mg./kg. i.v. | ++ | general | ++ | ++ |
| *41 | 0·5 mg./kg. i.v. | ++ | p. | +++ | ++ |

* Received 2 doses of ink at 0 and 30 minutes.

N.B. The doses of lysolecithin may have been less than stated as emulsification in saline was probably not complete.

as it was realised afterwards that the lysolecithin and the saline should have been titurated more thoroughly in a mortar until a milky solution was obtained.

### DISCUSSION

In the Majno, Palade and Schoefl technique Pelikan ink injected i.v. leaks through the endothelium of inflamed vessels but is arrested by the intact basement membrane thus outlining vessels where inflammatory exudation is occurring.

In this investigation we used this technique to answer two questions.

(1) Would the untreated tumours have so many factors producing inflammation that there would be widespread carbon marking of vessels?

(2) If mediators of the inflammatory reaction were injected into the circulation would the tumour vessels pick these up and become inflamed with doses which did not affect normal tissues?

The answer to problem one is that some vessels in the control tumours showed carbon labelling but the number of vessels labelled and the extent of the labelling was less in the control tumours than in the tumours of rats treated systemically with inflammatory agents. Also, in the control tumours marked vessels were frequently near to zones of necrosis, haemorrhage, polymorphonuclear infiltration or cysts. However, zones where tumour cells were actively infiltrating muscle often showed carbon labelled vessels.

The answer to problem two is that 5-HT (5 mg. base/kg., i.p.), bradykinin (2μg./kg./min., i.v. for 10 minutes), kallikrein (not less than 1 unit/kg., i.v.) and lysolecithin (not less than 2 mg./kg., i.v.) all increased the number of tumour vessels which showed carbon labelling. Pooling of blood in vessels of less than 15 μ diameter was also a common feature of the tumours of rats treated systematically with the inflammatory agents. Normal muscle of both control and treated animals showed virtually no marking of the vessels.

There were some differences in the effects produced by the different inflammatory agents. Thus 5-HT tended to produce carbon labelling of some vessels and pooling in others. Vessels showing pooling were usually unmarked. Evidence from measurements of oxygen tension in tumour and the response when the animal breathes oxygen often indicated complete circulatory stasis in tumours after this dose of 5-HT (Cater, Grigson and Watkinson, 1962; Cater, Schoeniger and Watkinson, 1963). Could this stasis be due to inflammatory exudation from the tumour vessels proceeding to the stage that the vessels become blocked by a solid mass of red cells, or did 5-HT produce stasis by causing constriction of arterioles and venules? We argued that if 5-HT stasis was due to exudation this would not be complete by the time one injection of Pelikan ink had been cleared from the circulation—about 30 minutes—and that a second dose of ink should increase the labelling. On the other hand if the 5-HT stasis was due to vasospasm then it was likely to be complete inside 30 minutes and a second dose of ink should not increase the number and extent of labelled vessels. In fact, we found that the second dose of ink did not increase the number of marked vessels. If our reasoning is correct this is in favour of vasospasm. Also some vessels with no ink against the basement membrane now showed ink mixed with the solid mass of red cells in vessels full of pooled blood. This is also in favour of 5-HT producing stasis by vasospasm. The evidence from the oxygen-tension measurements indicated that stasis had occurred within 20 minutes of injection of 5-HT and this is in favour of vasospasm.

The salient feature of bradykinin treated rats was the presence of carbon labelled venules (and some capillaries) in the middle of zones of dividing tumour cells, with cells in mitosis quite close to the marked vessels. Had the carbon labelled vessels been near to necrotic tumour the finding would have been of little significance, but finding labelled vessels in the zones of active growth argues in favour of the effect being real and important.

Kallikrein would be expected to produce results similar to bradykinin. Lewis (1964) and Miles (1964) have reviewed the activation of prokallikrein and its subsequent kininogenic activity. The inactive precursor of kallikrein, present in blood, is activated by contact with certain foreign substances, a sequence in which the Hageman factor has an acknowledged role. Activated kallikrein enzymatically hydrolyses an $\alpha 2$ globulin substrate to form plasma kinin which is closely similar to bradykinin, if not identical. Recent evidence suggests that several kinins are produced including bradykinin (Lewis, 1964). The kinins produced may include those which lyse clots and basement membranes.

Cotran and Majno (1964) distinguished four types of vascular leakage. (1) The immediate-type, coming on early in inflammation but transient, and the carbon labelling is predominantly venular. Histamine, 5-HT, bradykinin, permeability-factor and lactic acid all mimic the immediate response.

(2) The delayed-type, causing a prolonged exudation, and the carbon-labelling is mainly in the capillaries with some involvement of the arterioles and venules. The delayed response could be due to slow liberation of bradykinin and other kinins *in situ* by the action of kallikrein, but the capillary localisation is against this.

(3) Direct vascular injury following severe trauma which involves all types of vessels.

(4) Leakage from regenerating capillaries (Schoefl, 1964).

The effect of lysolecithin on tumour vessels was studied because Fischer and Haupt (1961) claimed that in some forms of injury complement activity may release lysolecithin. Cotran and Majno (1964b) injected lysolecithin into the cremaster muscle of the rat and found definite carbon labelling of the capillaries as well as of the venules, especially if the Pelikan ink was injected 2 hours after lysolecithin. They therefore think that lysolecithin might be an important factor in the delayed-type of inflammatory exudate. Fischer (1964) found that antigen/antibody complexes plus complement damaged the oxygen utilisation of ascites tumour cells. He assumed that the activated complement was equivalent to lysolecithin. Butterworth and Cater (as yet unpublished) have found that lysolecithin *per se* does damage the oxygen uptake of Ehrlich ascites tumour cells and BP8 ascites tumour cells. Thus lysolecithin could damage the tumour cells and the damage result in inflammatory changes, but it is much more likely that the carbon labelling observed was due to direct damage of the vascular endothelium by the lysolecithin. We also found some evidence suggestive of agglutination of red cells. It is possible that we would have obtained more labelling of the tumour vessels if we had injected the Pelikan ink 1 to 2 hours after the lysolecithin. The dosage/response effect of lysolecithin would repay further study.

It is pertinent to enquire why inflammatory agents given systemically should affect tumour vessels more than those of normal tissues. There are a number of possible explanations :

(1) Tumour vessels could be simply a special case of a general principle that all newly formed vessels are unduly susceptible to inflammatory agents. Schoefl (1964) found that the tips of new capillary buds leaked ink and in his experiments they had not been subjected to deliberate damage. In fact experiments are planned in which a direct comparison of tumour vessels with those in granulation tissue will be possible and their relative sensitivities to inflammatory agents studied.

(2) The damage of tumour vessels by inflammatory agents could be due to anatomical flaws inherent in their rapid formation. Algire and Chalkley (1945) watched sarcoma transplants grow in transparent chambers and found that tumour blood vessels originated from host capillary buds, as in granulation tissue, but their rate of growth far exceeded that in granulation tissue.

(3) The damage of tumour vessels by inflammatory agents could be due to the slow blood flow in dilated tumour vessels. The low oxygen tension in tumour argues in favour of low blood flow or high oxygen utilisation by the cells. The dye injection experiments of Goldacre and Sylvén (1959, 1962) and Owen (1960) indicate a poor blood flow especially in the central parts of tumours. The direct measurements by Gullino and Grantham (1961, 1962) of the blood flow in " tissue isolated transplants " indicated a very low blood flow in tumour. A low blood flow would allow time for the inflammatory agent to become localised. By comparison, in resting muscle a large proportion of the capillaries would be closed.

(4) Another possibility is that many of the tumour vessels are already in a state of subliminal inflammation, because of anoxia and the proximity of necrotic or damaged cells, and thus require only minimal quantities of inflammatory agents to produce demonstrable inflammatory exudation. This hypothesis would not explain the bradykinin effect where the labelled vessels were in good, actively growing tumour. It would only explain carbon labelling of vessels near some obvious cause of inflammation.

(5) The damage of tumour vessels by inflammatory agents could be due to certain conditions in tumour which produce vicious circles. We think these may be very important in tumours. For instance, the low oxygen tension often present in a tumour would inhibit the activity of mono-amine oxidase, if this enzyme were important in the destruction of 5-HT, then the destruction of 5-HT in tumour would be delayed even if the tumour contained a normal quantity of the enzyme. Or consider the case of bradykinin—this is quickly destroyed in the blood by kininases, but Edery and Lewis (1963) showed that at slightly lower pH values kininase is inhibited without any slowing of the production of kallikrein. The direct measurements of Voegtlin, Kahler and Fitch (1935) and Kahler and Robertson (1943) showed that tumours have a lower pH than normal tissues and that injection of glucose can lower this still further by production of lactic acid. Thus the destruction of bradykinin might well be slower in tumour than in normal tissue. This may also be true, to a greater or lesser extent, of granulation tissue.

The list of substances of physiological and pathological importance to which tumour vessels are abnormally sensitive now includes adrenaline, noradrenaline, acetyl choline (Cater, Adair and Grove, 1966), 5-HT, bradykinin, kallikrein, lysolecithin and Shear's polysaccharide (Shear, 1941 ; Shear and Perrault, 1944). This aspect of tumour physiology/pathology merits further investigation.

### SUMMARY

Rats with hepatomas transplanted in thigh were injected i.v. with Pelikan ink (colloidal carbon which labels inflamed vessels) and immediately afterwards either saline or an inflammatory agent was given systemically. The tumours of saline-injected control rats showed some labelled vessels situated peripherally and near necrotic or haemorrhagic zones. The inflammatory agents 5-HT, bradykinin, kallikrein and lysolecithin given systemically increased the labelling of vessels in zones of growing tumour. Muscle vessels were not labelled in controls or rats given inflammatory agents.

### REFERENCES

Algire, G. H. and Chalkley, H. W.—(1945) *J. natn. Cancer Inst.*, **6**, 73.
Cater, D. B., Adair, H. M. and Grove, C. A.—(1966) *Br. J. Cancer*, **20**, 504
Cater, D. B., Grigson, C. M. B. and Watkinson, D. A.—(1962) *Acta Radiol.* **58**, 401.
Cater, D. B., Schoeniger, E. L. and Watkinson, D. A.—(1963) *Acta Radiol. Ther. Phys. Biol.*, **1**, 233.
Cotran, R. S. and Majno, G.—(1964*a*) *Am. J. Path.*, **45**, 261.—(1964*b*) *Ann. N.Y. Acad. Sci.*, **116**, 750.
Edery, H. and Lewis, G. P.—(1963) *J. Physiol., Lond.*, **169**, 568.
Fischer, H.—(1964) *Ann. N.Y. Acad. Sci.*, **116**, 1063.
Fischer, H. and Haupt. I.—(1961) *Z. Naturf.*, **16B**, 321.
Goldacre, R. J. and Sylvén, B.—(1959) *Nature, Lond.*, **184**, 63.—(1962) *Br. J. Cancer.*, **16**, 306.
Gullino, P. M. and Grantham, F. H.—(1961) *J. natn. Cancer. Inst.*, **27**, 1465.—(1962) *J. natn. Cancer. Inst.*, **28**, 211.
Kahler, H. and Robertson, W.—(1943) *J. natn. Cancer Inst.*, **3**, 495.
Lewis, G. P.—(1964) *Ann. N.Y. Acad. Sci.*, **116**, 847.
Majno, G., Palade, G. E. and Schoefl, G. I.—(1961) *J. biophys. biochem. Cytol.*, **11**, 607.
Miles, A. A.—(1964) *Ann. N.Y. Acad. Sci.*, **116**, 855.
Owen, L. N.—(1960) *Nature, Lond.*, **187**, 795.
Schoefl, G. I.—(1964) *Ann. N.Y. Acad. Sci.*, **116**, 789.
Shear, M. J.—(1941) *Cancer. Res.*, **1**, 731.
Shear, M. J. and Perrault, A.—(1944) *J. natn. Cancer, Inst.*, **4**, 461.
Voegtlin, G., Kahler, H. and Fitch, R. H.—(1935) *Natn. Inst. Hlth Bull.*, **164**, 15.
Willis, R. A.—(1953) ' Pathology of Tumours ', London (Butterworth), p. 134.